# Hydrogen-rich saline attenuates eosinophil activation in a guinea pig model of allergic rhinitis via reducing oxidative stress

**DOI:** 10.1186/s12950-016-0148-x

**Published:** 2017-01-13

**Authors:** Shaoqing Yu, Chuanliang Zhao, Na Che, Lin Jing, Rongming Ge

**Affiliations:** Department of Otolaryngology, Tongji Hospital, Tongji University, 389 Xincun road, Putuo District, Shanghai, 200065 China

**Keywords:** Allergic rhinitis, Reactive oxygen species, Hydrogen-rich saline, Eosinophil, Eosinophil cationic protein, Eotaxin, Guinea pig

## Abstract

**Background:**

It is well considered that reactive oxygen species (ROS) plays a prominent causative role in the development of allergic rhinitis (AR), and eosinophils cells as important allergic inflammatory cells contribute to elevating oxidative stress. Hydrogen, emerging as a novel antioxidant, has been proven effective in selectively reducing ROS in animals models of oxidative damage. We herein aim to verify protective effects of hydrogen on eosinophils cells in guinea pigs models of AR.

**Methods:**

Thirty two guinea pigs were random divided into four groups, and AR model was established through ovalbumin sensitization. The guinea pigs were injected with hydrogen-rich saline (Normal-HRS and AR-HRS group) or normal saline (control and AR group). The frequencies of sneezing and scratching were recorded. The IgE level, blood eosinophil count and eosinophil cationic protein (ECP) level in serum were measured. The serum malondialdehyde (MDA) and superoxide dismutase (SOD) assays were also measured to evaluate oxidative stress. The expression levels of eotaxin mRNA and protein in the nasal mucosa were also determined by real-time RT-PCR, Western blot and immunofluorescence.

**Results:**

HRS reduced the ROS and MDA levels and increased SOD level in guinea pigs of AR-HRS group accompanied with decreased frequency of sneezing and scratches. Meanwhile, there was a decline of the number of eosinophils cells in blood and of thelevel of ECP in serum in the AR-HRS group. HRS also significantly decreased the expression of eotaxin in nasal mucosa.

**Conclusion:**

HRS may play a protective role in attenuating allergic inflammation, and suppressing the increase and activation of eosinophils in AR possibly through antioxidation effect of hydrogen.

## Background

Rhinitis, especially allergic rhinitis (AR), is a major health problem. Although several treatments such as corticosteroid and anti-histamine drugs are available for dealing with it, their side effects have limited the use of them. Recent studies have shown that oxidative stress and the production of reactive oxygen species (ROS) contribute to allergic inflammation, such as asthma and AR. The main sources of ROS are NAD (P) H oxidase, xanthine oxidase, lipoxygenase, mitochondria, and the uncoupling nitric oxide synthase [1]. It has been reported that the oxidation ability is significantly increased in patients with atopic asthma and AR compared to healthy individuals [2].

Many observations suggest that oxidative stress plays an important role in the pathogenesis of airway allergic inflammation such as asthma and AR [3]. As important pathogenic factors of AR, ROS can increase mucosa permeability and mucus production accompanied with influx of inflammatory cells, decrease numbers and function of epithelial cilia, and alter expression of adhesion molecules and release of inflammatory mediators. A growing number of reports havedemonstrated that oxidative stress and its resultant production of ROS play prominent roles in the mechanism of inflammatory responses during asthma and AR [4–6].

Airway and circulating inflammatory cells and especially eosinophils are the likely source of ROS increases. For instance, antigen challenge increases spontaneous ROS from airway eosinophils in patients with asthma [7], and some research shows that blood eosinophils also produces more ROS in asthmatic patients compared with control subjects [8]. The eosinophils isolated from allergic patients also produces more hydrogen peroxides when challenged with antigen [9]. Thus eosinophils cells as important allergic inflammatory cells contribute to elevated oxidative stress in allergic disease.

Recently, it has been proved that hydrogen gas, a highly flammable gas, has potent antioxidant property. Compared with other antioxidants gas such as carbon monoxide (CO) and Hydrogen sulfide (H_2_S), hydrogen could selectively reduce the hydroxyl radical (∙OH) and peroxynitriteanion (ONOO-), the most cytotoxic chemicals of ROS, and effectively protect cells; in addition, hydrogen does not react with other ROS, which possesses physiological roles [10]. Since the hydrogen molecule is electrically neutral and much smaller than the other antioxidants, it is able to easily penetrate membranes and enter cells and organelles, such as the nucleus and mitochondria, where most commonly used antioxidants cannot arrive [11]. However, hydrogen gas inhalation as a clinical application is not convenient and may be dangerous because it is inflammable and combustible. Compared with hydrogen gas, hydrogen saturated in saline (i.e., a hydrogen-rich saline, HRS) is safe and easy to administer. It has been demonstrated that treatment with HRS protects organs damage from oxidation effect in animals models such as transient cerebral ischemia [12], neonatal cerebral hypoxia–ischemia [13], renal injury [14] and myocardial injury induced by ischemia and reperfusion [15]. Although some study shows that HRS as an interesting anti-allergic treatment could alleviate respiratory symptom of asthma [16], no researches focus on the effect of treatment with hydrogen on AR, so we make the present study to explore whether HRS could, in its potential as an antioxidant for preventive and therapeutic applications in AR, attenuate eosinophil activation through guinea pigs models.

## Methods

### Material and animal models

HRS was generously offered by Professor Li Bailong (the Second Military Medical University, Shanghai, China) and prepared as described previously [17]. In short, hydrogen gas (0.4 mPa) was dissolved in normal saline for at least 2 h to reach supersaturated level (>0.6 mmol/L).

Mature healthy male guinea pigs weighing ±230 g were purchased from National Rodent Laboratory Animal Resources (Shanghai, China). All animal care and experimental procedures were approved by the Tongji University Institution Animal Care and Use Committee (2014-DW-009).

The AR models were prepared as follows. 32 guinea pigs were randomly divided into four groups (*n* = 8 each group), namely, Control groups, normal-HRS, AR-NS and AR-HRS. The AR-NS and AR-HRS groups were both sensitized with ovalbumin (OVA, Sigma Corporation, USA). Each guinea pig was first sensitized intraperitoneally with 0.3 mg of OVA and 30 mg of Aluminum hydroxide (AL (OH) 3) every other day for a total of seven times. From day 15 on, the guinea pigs were treated with 0.5% OVA aerosol to stimulate AR symptoms for five times. Subsequently, each side of the nasal cavity was given 20 μL of 2% OVA solution intranasally once a day to maintain AR symptoms. The control group and normal-HRS groups were health guinea pigs who are given the same dose of saline treatment.

From day 20 on, the guinea pigs of the AR-HRS group and Normal-HRS group were intraperitoneally injected with a dose of 10 mL/kg HRS and intranasally with 20 μL of HRS on each side once a day. The guinea pigs in the AR-NS group and control group were also given the same dose of saline both intraperitoneally and intranasally every day. The treatment continued for 14 days.

### Observation of frequencies of scratching and sneezing

The frequencies of scratching and sneezing were assessed for 1 h directly after nasal challenge. Sneezing was characterized by an explosive expiration just after deep inspiration. Scratching was characterized by an external perinasal scratch with the animal’s forelimbs.

### Determination of total IgE and eosinophil cationic protein (ECP) level in serum

The guinea pigs were anesthetized by intraperitoneal administration of pentobarbital (40 mg/kg). These animals were sacrificed by rapid decapitation, and then blood and nasal mucosa were collected. Biopsies of the nasal mucosa were taken from the inferior turbinate and immediately placed in liquid nitrogen. Peripheral blood was collected and the separated serum was kept at − 20 °C. Serum total IgE (R&D Corporation,USA) and ECP (R&D Corporation, USA) were determined by enzyme-linked immunosorbent assay method (CAP system). The detectable range of the assay for total IgE is 2–200 ng/mL, and for serum ECP is 2 to 100 ng/L.

### Blood eosinophil count

A peripheral venous blood sample was collected and eosinophils were counted through automatic blood cell analyzer (Sysmex Medical Electronics Co. Ltd. Japan). The detectable range of blood eosinophil counted in the same way is 0 to 10,000/μL.

### Determination of ROS in serum and nasal mucosa

The levels of serum ROS were measured with highly sensitive enzyme-linked immunosorbent assay (ELISA) kits according to the manufacturer’s recommendations (Shanghai Tongwei Co., Ltd, China). The absorbance (OD) was measured at 450 nm wavelength with the enzyme marker, and the ROS concentration in the sample was calculated by standard curve. The detectable range of the assay is 10 IU/ml -320 IU/ml.

ROS levels in nasal mucosa were measured with 2,7-dichlorofuorescin diacetate (DCFH-DA). Collected nasal mucosa were incubated with DCFH-DA for 30 min at 37 °C. DCFH-DA forms a fluorescent product, DCF (dichloro fluorescein) upon oxidation with ROS. Fluorescence caused by DCF in each well was measured and recorded for 30 min at 500 nm (excitation) and 530 nm (emission).

### Serum malondialdehyde (MDA) and superoxide dismutase (SOD) assays

To further confirm that HRS-induced inhibition of inflammation was related to the anti-oxidative property of HRS, the oxidative stress markers were measured with MDA and SOD. SOD is endogenous antioxidants as free radical scavenger, and MDA is the lipid per oxidation end product.

Serum MDA levels were measured with a commercial MDA assay kit (Nanjing Jiancheng Bioengineering Institute, Nanjing, China). Briefly, hydroxytoluene combined with thiobarbituric acid was able to become red. The absorbance of condensation products was tested at a wavelength of 532 nm. The levels of MDA in nasal mucosa tissue were normalized against total protein (mg protein/mL). The activity of SOD was measured with a commercial assay kit (Nanjing Jiancheng Bioengineering Institute, Nanjing, China), in accordance with the manufacturer’s instructions. Briefly, this assay kit uses a thiazole salt for detection of superoxide anions to produce a colored product; absorbance was tested at a wavelength of 450 nm.

### RNA isolation and real-time RT-PCR for eotaxin

Fluorescent quantitative real time RT–PCR was performed to determine the expression levels of eotaxin mRNAs in nasal mucosa. The total RNA was extracted and cDNA was synthesized with a cDNA synthesis kit (Prime Script RTase, TaKaRa Inc., Japan). To determine the expression of eotaxin, we performed a fluorescent quantitative real-time RT–PCR assay. Primer sequences used are listed in Table 1; mRNA levels were normalized relative to GAPDH mRNA levels.

**Table 1:** Examined genes and their PCR primers

Gene	Primer sequence 5′ → 3′	Amplification size (bp)
eotaxin-1	F:5′-AACCCAGAAACTATTGTCACGCT-3′ R:5′-GGACATTTGCTGCTGGTGATTAT-3′	216
GAPDH	F:5′-AAAGGCATCTTGGGCTACACC-3′ R:5′-GCTGTAGCCGAACTCATTGTCATA-3′	153

### Western blot analyses of eotaxin

The guinea pig nasal mucosa (10 mg), which was frozen in liquid nitrogen, was homogenized in 1 mL of protein lysis buffer (PBS containing 0.1% Triton X-100) and centrifuged at 14,000 g for 10 min at 4 °C. Nasal mucosa lysates from each group were analyzed by Western blot. The blots were blocked with PBS-T containing 1% skim milk and then incubated with a 1:1000 dilution of anti-eotaxin antibody (Beyotime biotechnology, China) at room temperature. After three additional washes, the blots were incubated with anti-mouse secondary antibody (1:5000) (Beyotime biotechnology, China) and then conjugated to horseradish peroxidase for 1 h at room temperature. The second batch of images was quantified using Quantity One software (Bio-Rad Laboratories, USA). GAPDH was employed as an endogenous control for protein normalization. The experiments were performed in duplicate.

### Immunofluorescence of eotaxin in nasal mucosa

Tissues from the specimens were fixed in 10% buffered formalin. Immunohistochemical stains were performed on formalin-fixed and paraffin-embedded 4 μm sections. The tissue sections were deparaffined, and permeabilized in precooled acetone at − 20 °C for 15 min. Slides were washed in PBS. Normal goat serum (10% in PBS) was used to block nonspecific antibody binding. The slides were incubated for 1 h at room temperature with anti–Eotaxin-1 rat monoclonal antibody (1:100, Boster Co. Ltd. China). Slides were washed twice in PBS, and then incubated for 30 min at room temperature with anti-rat Cy3-conjugated IgG (1:50, Boster Co. Ltd. China). Slides were washed twice in PBS and once in distilled water and allowed to dry. Fluorescence microscope was applied to observation and photograph.

### Statistical analysis

Statistical analyses were performed using SPSS version 17.0 (SPSS Inc., Chicago, Illinois, USA). All data were expressed as mean ± S.D. Statistical analyses of data were performed using ANOVA for multiple comparison and Least Significant Difference (LSD) for comparison among groups, and Pearson Correlation for the two-variable correlation analysis. *p* < 0.05 was considered statistically significant.

## Results

### Frequencies of scratching and sneezing and IgE level in serum

**Fig. 1:**
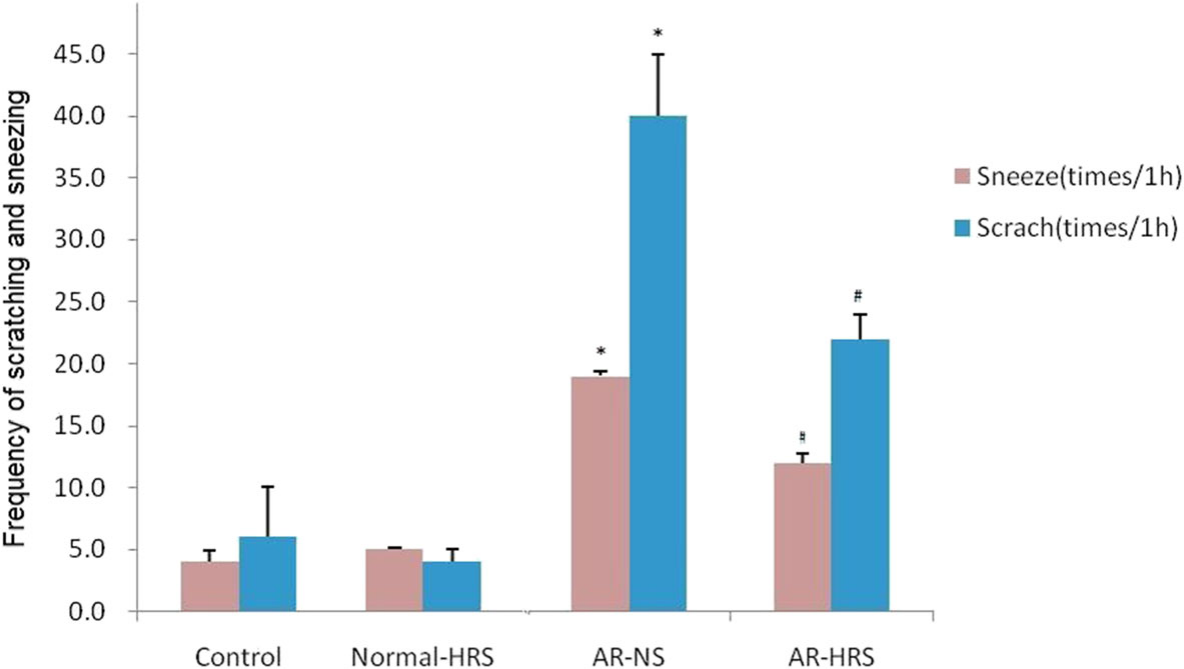
Frequency of scratching and sneezing of guinea pigs. For the four groups: saline control, normal-HRS, AR- NS and AR-HRS. Each column and vertical bar represents the mean ± S.D. *,**: Significantly different from the control group (*p* < 0.05 and *p* < 0.01, respectively). ^#^,^##^ Significantly different from the AR-NS group (*p* < 0.05 and *p* < 0.01, respectively)

The frequencies of scratching and sneezing are shown in Fig. 1. The frequencies of scratching and sneezing in the AR-sensitized group were significantly increased compared with the control group (*p* < 0.05), and no significant changes were found in normal guinea pigs after HRS treatment (*p* > 0.05). In the AR-HRS group, the frequencies of scratching and sneezing decreased significantly compared with those of the AR-NS group (*p* < 0.05). It indicates that inflammatory symptom of AR was alleviated by HRS in guinea pigs.

**Fig. 2:**
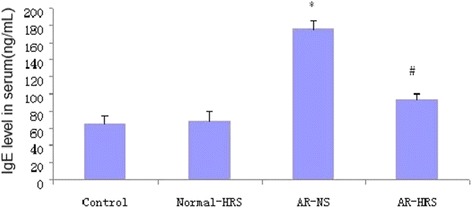
IgE level in serum of guinea pigs. For the four groups: saline control, normal-HRS, AR- NS and AR-HRS. Each column and vertical bar represents the mean ± S.D. *, **: Significantly different from the control group (*p* < 0.05 and *p* < 0.01, respectively). ^#^, ^##^ Significantly different from the AR-NS group (*p* < 0.05 and *p* < 0.01, respectively)

The level of IgE of groups is shown in Fig. 2. In the AR-NS group it is higher than that in the control group (175.44 ± 10.02 ng/mL vs.65.24 ± 8.44 ng/mL, *p* < 0.01). After HRS treatment, the IgE content in the AR-HRS guinea pigs is significantly decreased (92.70 ± 7.66 ng/mL vs 175.44 ± 10.02 ng/mL, *p* < 0.05). Otherwise, there is no significant difference between control and Normal-HRS group (68.76 ± 10.45 g/mL vs.65.24 ± 8.44 ng/mL, *p* > 0.05). All these results indicate that HRS could reduce the inflammatory response of AR accompanied with decreased IgE level.

### HRS treatment reduced oxidative stress

**Fig. 3:**
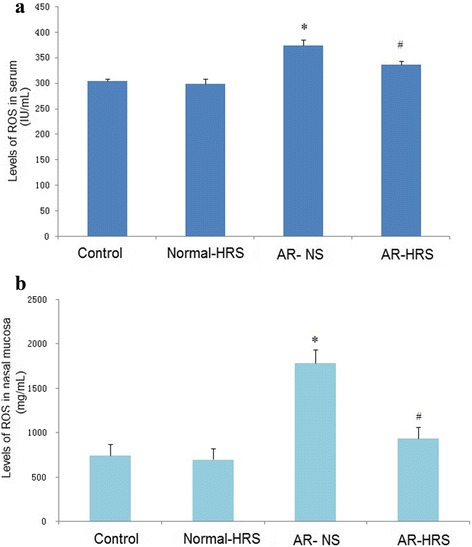
Levels of ROS in serum and nasal mucosa of groups. For the four groups: saline control, normal-HRS, AR- NS and AR-HRS. Levels of ROS in serum (**a**) and nasal mucosa (**b**) were shown. Each column and vertical bar represents the mean ± S.D. *,**: Significantly different from the control group (*p* < 0.05 and *p* < 0.01, respectively). ^#^,^##^ Significantly different from the AR-NS group (*p* < 0.05 and *p* < 0.01, respectively)

**Fig. 4:**
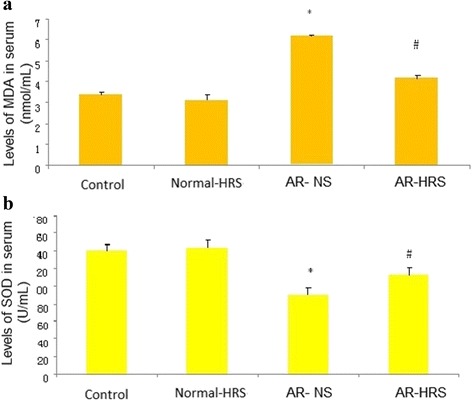
Levels of MDA and SOD in serum of groups. For the four groups: saline control, normal-HRS, AR- NS and AR-HRS. Levels of MDA (**a**) and SOD (**b**) in serum were shown. Each column and vertical bar represents the mean ± S.D. *,**: Significantly different from the control group (*p* < 0.05 and *p* < 0.01, respectively). ^#^,^##^ Significantly different from the AR-NS group (*p* < 0.05 and *p* < 0.01, respectively)

**Fig. 5:**
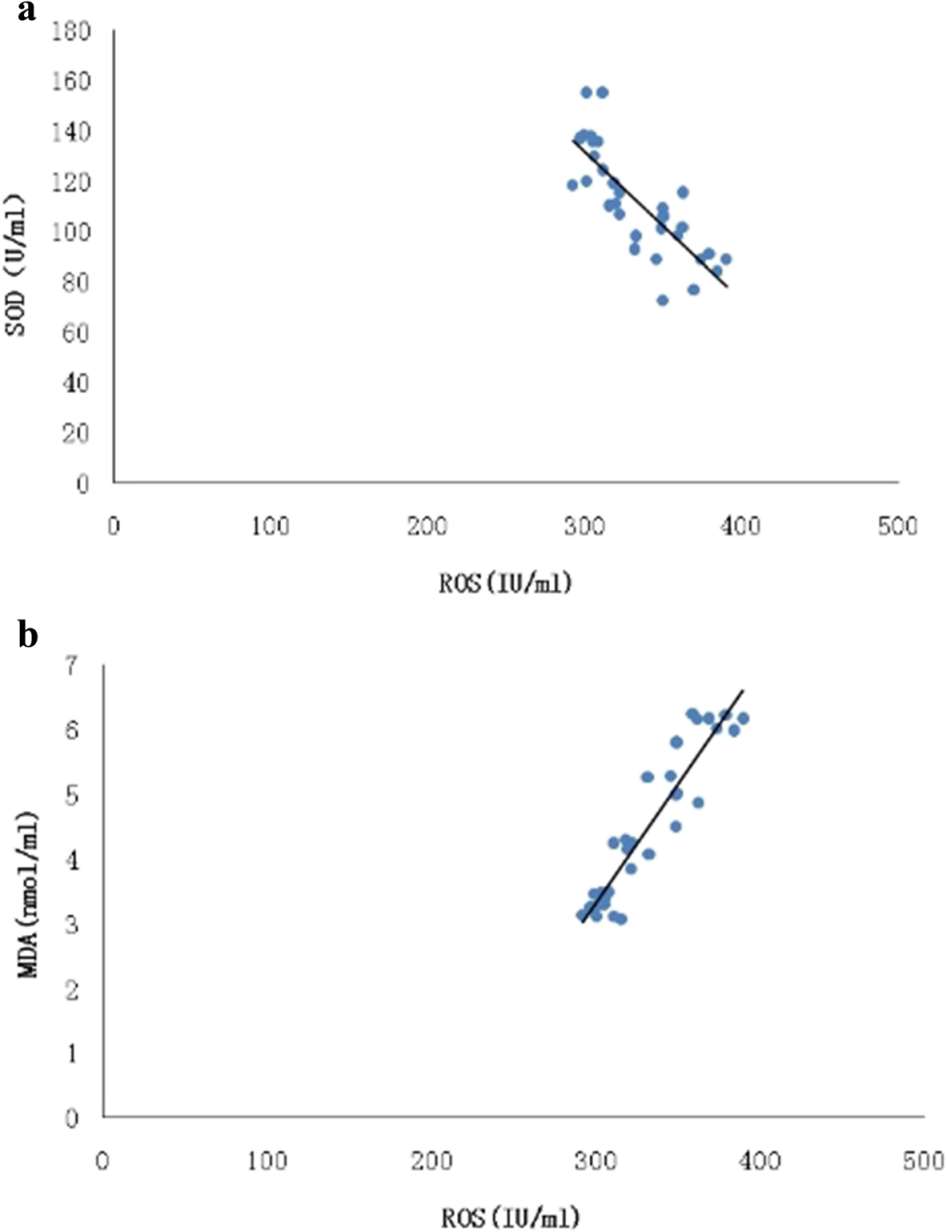
Correlation analysis of serum ROS, MDA and SOD. Serum ROS was correlated with MDA levels (*r* = 0.87, *P* < 0.01) (**a**), and SOD levels (*r* = 0.64, *P* < 0.01) (**b**)

Enhanced oxidative stress was observed in AR animals, as evidenced with the elevated ROS levels of serum and nasal mucosa (Fig. 3a and b), and the elevation appeared to be significantly inhibited by HRS treatment (*p* < 0.05). Moreover, decreased changes for SOD activity and increased MDA levels in serum were observed (Fig. 4a and b), and after HRS treatment, elevated ROS was inhibited by HRS accompanied with increased SOD level and decreased MDA level significantly (*p* < 0.05). Similarly, no significant difference between control and Normal-HRS group was found (*p* > 0.05). The correlation between the makers was also carried out, and there was a highly significant correlation between ROS and MDA levels (*r* = 0.87, *P* < 0.01) (Fig. 5a), suggesting that levels of MDA were positively correlated with ROS. Otherwise, there was also a highly significant correlation between ROS and SOD levels (*r* = 0.64, *P* < 0.01) (Fig. 5b), suggesting that levels of SOD were negatively correlated with ROS. All this suggests that HRS treatment could reduce oxidative stress of AR significantly.

### Blood eosinophil count and ECP concentrations in serum

**Fig. 6:**
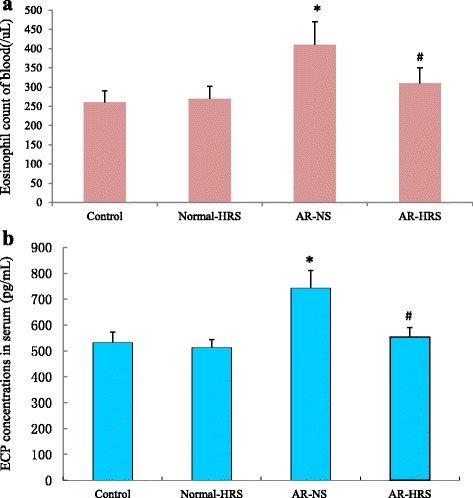
Blood eosinophil count and ECP concentrations in serum of groups. For the four groups: saline control, normal-HRS, AR- NS and AR-HRS. Blood eosinophil count and ECP concentrations in serum of groups were shown. Each column and vertical bar represents the mean ± S.D. *,**: Significantly different from the control group (*p* < 0.05 and *p* < 0.01, respectively). ^#^,^##^ Significantly different from the AR-NS group (*p* < 0.05 and *p* < 0.01, respectively)

Similarly, eosinophil count in AR group was significantly higher than in control group. ((0.41 ± 0.06) × 10^9^/L vs.(0.26 ± 0.03) × 10^9^/L, *p* < 0.05) and decreased significantly with HRS treatment ((0.31 ± 0.04) × 10^9^/L, *p* < 0.05) (Fig. 6a). Otherwise, no significant difference was also found between control group and normal-HRS groups ((0.32 ± 0.03) × 10^9^/L vs. (0.26 ± 0.03) × 10^9^/L, *p* > 0.05).

**Fig. 7:**
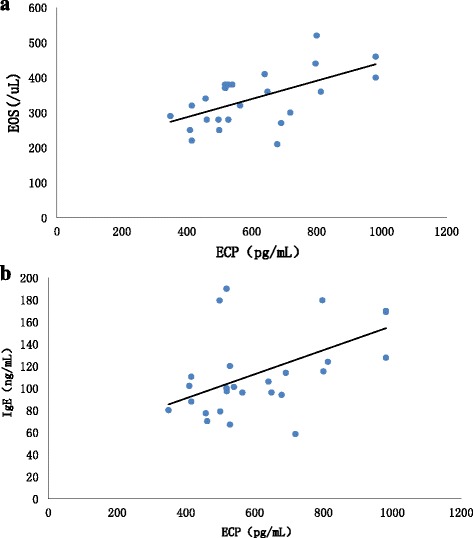
Correlation analysis of serum ECP, eosinophil count and serum total IgE. Pearson Correlation was used to analyze the relationship. Serum ECP was correlated with blood eosinophil count (*r* =0.65, *p* < 0.01) (**a**), and serum total IgE (*r* = 0.56, *p* < 0.01) significantly (**b**)

Serum ECP level was 741.98 ± 69.7 pg/mL in AR group was significantly higher than that of control subjects (533.13 ± 39.14 pg/mL, *p* < 0.05), while it was 554.48 ± 36.53 pg/mL in AR-HRS group and 512.39 ± 32.478 in normal-HRS group (Fig. 6b). There was a significantly difference between AR-HRS and AR-NS groups (*p* < 0.01). Also, serum ECP was correlated with blood eosinophil count (*r* = 0.65, *p* < 0.01) (Fig. 7a), and serum total IgE (*r* = 0.56, *p* < 0.01) significantly (Fig. 7b).

### Reduction of eotaxin mRNA and protein expression in nasal mucosa by HRS treatment

**Fig. 8:**
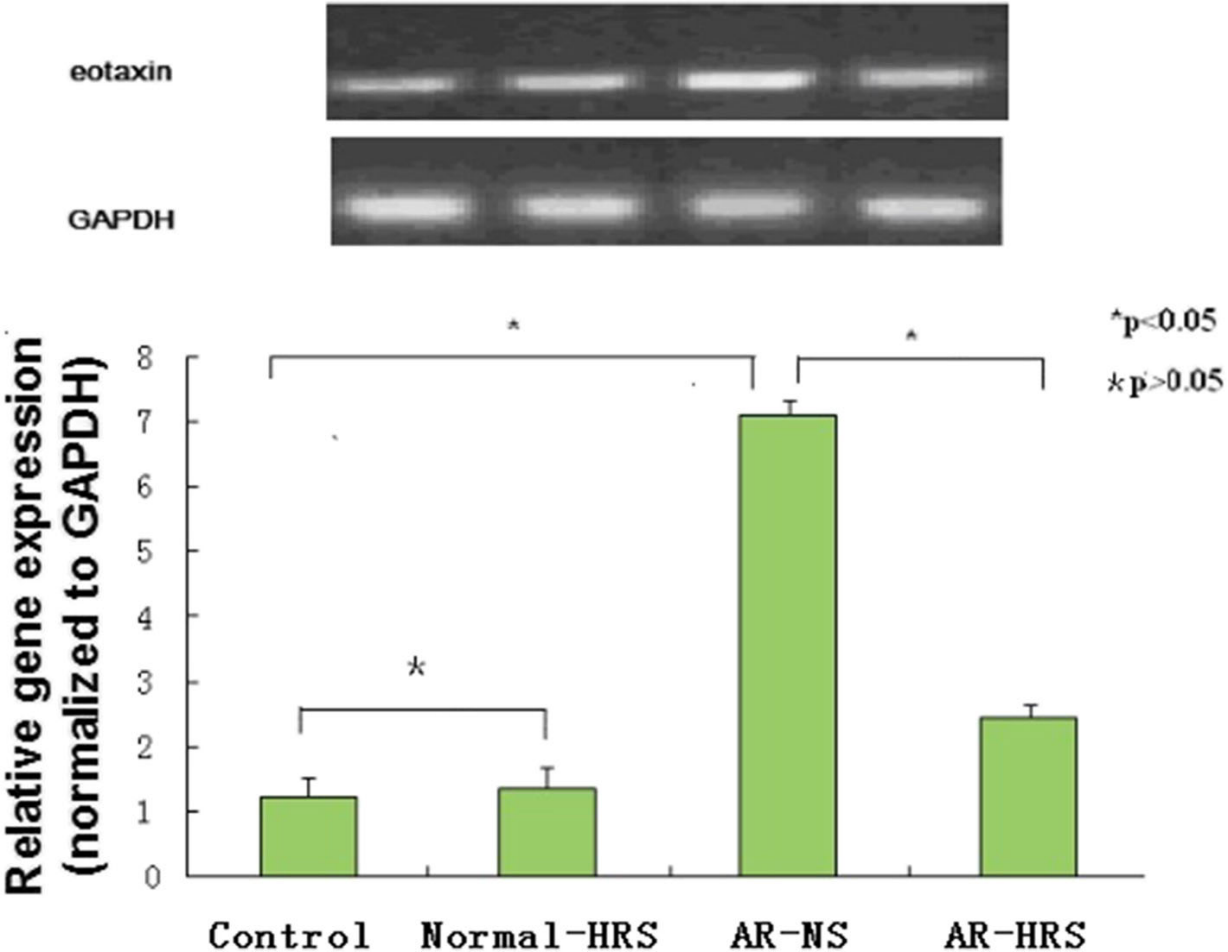
Expression of eotaxin mRNA in nasal mucosa. Sizes of PCR products are 216 bp (eotaxin) and 153 bp (GAPDH). Lanes from left to right were products of saline control, normal-HRS, AR-NS and AR-HRS. There was an increase in eotaxin mRNA in AR-NS group compared with control, and eotaxin decrease dafter HRS treated (*p < 0.05 versus control, #*p* < 0.05 versus AR-NS), whereas there was no change in GAPDH mRNA. No significance of mRNA expression were shown between control and Normal-NS groups (*p > 0.05)

**Fig. 9:**
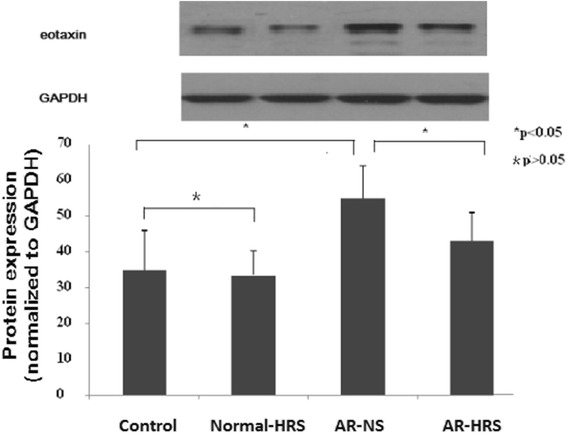
Western blot analysis of eotaxin protein in groups. For the four groups: saline control, normal-HRS, AR- NS and AR-HRS. Western blot analysis showed that the relative expression of eotaxin protein in the AR-NS group was much higher than that in control group, and decreased in AR–HRS group, (*p < 0.05 versus control, #*p* < 0.05 versus AR-NS). No significance of protein expression were shown between control and Normal-NS groups (*p > 0.05)

The eotaxin mRNA and protein expression in the nasal mucosa of the AR sensitized significantly grew up (*p* < 0.05). Similarly, no significant difference between control and Normal-HRS group was found (*p* > 0.05). However, the mRNA and protein expression of eotaxin in the HRS-treated group decreased significantly compared with that of the AR-NS group (*p* < 0.05 for each (Figs. 8 and 9). These results indicate that HRS could suppress the eotaxin expression in allergic inflammation conditions.

### Assessing eotaxin expression by immunofluorescence

**Fig. 10:**
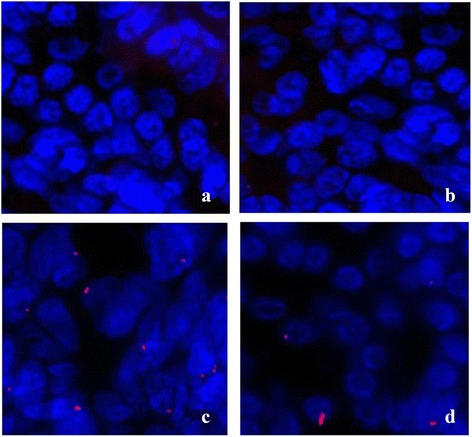
Immuno cytochemistry of Eotaxin in groups. Eotaxin expression in control, AR-NS, AR-HRS and Normal-HRS groups was determined by immuno cytochemistry. Immunoreactive eotaxin, detected using a Cy3-labeled secondary antibody (*red*), was highly expressed in nasal mucosa of AR-NS group (**c**), and decreased in AR-HRS group (**d**). While in control subjects and normal-HRS subjects eosinophil infiltration was very scarce (**a** and **b**)

Histology seems to confirm the above findings. Figure 10c shows plenty of eotaxin in the cell nucleus of nasal mucosa taken from AR and NS group, while in control subjects and normal-HRS subjects eosinophil infiltration is very scarce (Fig. 10a and b). Otherwise, the eotaxin of the HRS-treated group is relatively low expression (Fig. 10d). All this indicates that HRS could reduce eotaxin expression in the nasal mucosa of AR.

## Discussion and conclusions

Our results have demonstrated that HRS, functioning as a scavenger of ROS, could have the anti-allergic inflammation therapeutic value in AR disease. ROS are classically defined as partially reduced metabolites of oxygen that possess strong oxidizing capabilities. They contain ∙OH, O2, H2O2, ONOO, NO−, are important cytotoxic molecules and signal mediators in the pathophysiological mechanisms of inflammatory diseases [18, 19]. Among them, ∙OH and ONOO− are much more reactive than others and are regarded as major cytotoxic mediators of cellular oxidative damage [20].

Under physiological condition, tissues contain various endogenous antioxidant enzymes like GSH and SOD, which scavenge ROS and prevent lipid peroxidation, and maintain the relevant balance [21]. In the process of AR, the antigens have been shown to induce human nasal epithelial cells to produce ROS that trigger cytokine production in AR, and ROS were overproduced and then induced an imbalance between the ROS and endogenous antioxidants or antioxidant enzymes. The overproduced ROS were able to directly or indirectly damage the nasal mucosa, which results in mucosa dysfunction and histological changes [22].

In 2007, researchers from Japan reported that hydrogen gas possessing antioxidant and anti-apoptotic properties could protect the brain against ischemia-reperfusion injury and stroke by selectively reducing hydroxyl radicals · OH and ONOO-in cell-free systems. Indeed, more researches have been reported that hydrogen reacts only with the strongest oxidants (∙OH and ONOO−) and does not disrupt metabolic redox reactions or ROS concerned in cell signaling [23]. It is demonstrated that HRS has an essential role in ROS clearance. In vitro study, the researchers demonstrated that hydrogen showed promising efficacy in many disease models [24, 25]. A growing number of reports have demonstrated that oxidative stress and its resultant production of ROS play prominent roles in the mechanism of inflammatory responses as asthma, renal injury, etc. In the process of inflammation, ROS are overproduced and then induce an imbalance between the ROS and endogenous antioxidants or antioxidant enzymes, and HRS can neutralize the ROS and eases the oxidative damage in inflammation such as asthma and pancreatitis [16, 26]. However, there are no reports of HRS in research of AR disease, although HRS may also exert anti-inflammatory effects in rhinitis.

In order to observe the antioxidation effect of HRS in AR, levels of MDA and SOD in serum were observed in this experiment. MDA can be used as a crosslinking agent to promote the cross-linking of nucleic acids, proteins and phospholipids, and to change the function of biological macromolecules. The content of MDA can reflect the degree of lipid per oxidation. SOD, as disproportionation of superoxide anion generation of H2O2, can scavenge ROS, prevent lipid per oxidation, and protect cells from damaging of toxic oxygen radicals. Therefore, SOD is an important enzyme in the defense of the superoxidion from the body or external environment. The increase of MDA levels and decrease of SOD activity can lead to oxidative stress reaction, which will result in cell damage and even cell death. It is showed that in the process of allergen-induced airway inflammation, the inflammatory mediators can stimulate eosinophils to produce ROS that can be inhibited by SOD [27]. In this study, upregulated oxidative metabolism in allergic models compared to those from normal subjects was shown, and ROS levels in AR animal appeared to be reduced by HRS accompanied with increased SOD level and decreased MDA level. These results indicate that HRS neutralizes the ROS and eases the oxidative damage from allergic inflammation. After HRS treatment, the AR guinea pigs showed relieved symptoms of allergic reaction, such as nasal scratching nose and sneezing. The observations indicate that HRS exerts potent anti oxidative and anti-inflammatory effects, and it can attenuate the severity of AR in guinea pigs.

In order to investigate the influence of HRS on inflammation of AR, the function and counts of eosinophils were also studied, because abnormalities of quantity and activity of eosinophils are typical features of AR. The present observations indicate that the declined number of eosinophils may be associated with drug-induced eosinophil apoptosis of tissue. It has been widely assumed that apoptosis of eosinophils of airway tissue would be increased by treatment such as corticosteroid, and inducement of eosinophil apoptosis has thus been advocated as a major pharmacological mechanism to bring about resolution of allergic inflammation [28, 29]. However, more studies are needed to confirm the induction of apoptotic eosinophils with HRS.

It has been known that ECP is released by activated eosinophils, and ROS with activated eosinophils affect the ECP levels [30]. Our study had shown that eosinophils concentration and ECP levels in serum is increased in guinea pigs with AR, and the ECP levels in AR animal appeared to be reduced by HRS accompanied with decreased eosinophils concentration in serum. The ECP levels in the serum could reflect the rate of activation of circulating eosinophils [31]. Indeed, the serum levels of ECP in allergic asthma and atopic dermatitis are significantly higher [32]. Since ECP may be a major cause of chronic airway inflammation, it may also be an objective parameter of inflammation in respiratory allergic diseases. It is strongly involved in upper and lower airway inflammation, such as AR, nasal polyps and widely used as a standard marker of eosinophil activity [33]. ECP levels, correlated well with the number of eosinophils, could be useful in evaluating the degree of inflammation in patients with AR, and as an effective clinical test for evaluating the effectiveness of allergic inflammation therapy [34], for eg. pollen specific immunotherapy [35]. In our research, the therapy with HRS in AR can influence serum ECP levels, which indicates that HRS can suppress active eosinophil in AR.

Moreover, it is reported that eotaxin has a greater effect on priming eosinophil ROS, and eotaxin plays an important role in the pathogenesis of allergic inflammation through eosinophil activation by priming the eosinophil oxidative metabolism [36]. In the present study, influence of HRS on eotaxin expression has also been studied. Eotaxin, which is a CC Chemokine, was originally isolated from the bronchoalveolar fluid of allergic guinea pigs [37]. It has a strong chemotactic activity that is specific for eosinophils, while it induced monocyte chemotaxis only at a high concentration, and did not induce neutrophil or lymphocyte chemotaxis at all [38]. The eotaxin regulates eosinophil trafficking during allergic inflammation and plays important role in allergic reaction. As shown in the results, it is an increase in ROS production with higher expressed eotaxin in nasal mucosa of AR models, and it is also indicated that ROS had an effect on the expression of eotaxin. Therefore, the expression of eotaxin was also decreased along with ROS production suppressed by HRS treatment. It is implied that HRS also can suppress the eotaxin expression and it may be related to a decrease in ROS production in nasal mucosa of AR models.

Our study was the first to evaluate the molecular hydrogen protects cells and tissues against oxidative stress in AR models. It also raised the possibility of treating allergic respiratory diseases by using medical gas as antioxidant therapy. Antioxidants gas, such as CO,H_2_S and H_2_, will be a new treatment strategy in the therapy of AR [39].

In conclusion, our data demonstrate that HRS attenuates airway inflammation and suppresses the function and counts of eosinophils in AR possibly through antioxidation effect of hydrogen. However, more investigations should be carried out to identify the underlying mechanisms in hydrogen-mediated anti-allergic inflammation in future. We also propose that molecular hydrogen could be widely used in medical applications as a safe and effective protective drug with minimal side effects.
